# Adolescent Congenital Central Hypoventilation Syndrome: An Easily Overlooked Diagnosis

**DOI:** 10.3390/ijerph182413402

**Published:** 2021-12-20

**Authors:** Marta Ditmer, Szymon Turkiewicz, Agata Gabryelska, Marcin Sochal, Piotr Białasiewicz

**Affiliations:** Department of Sleep Medicine and Metabolic Disorders, Medical University of Lodz, 92-215 Lodz, Poland; marta.insmk@gmail.com (M.D.); szymon.turkiewicz@stud.umed.lodz.pl (S.T.); sochalmar@gmail.com (M.S.); piotr.bialasiewicz@umed.lodz.pl (P.B.)

**Keywords:** CCHS, diagnosis, late-onset

## Abstract

Congenital central hypoventilation syndrome (CCHS), also known as Ondine’s curse, is a rare, potentially fatal genetic disease, manifesting as a lack of respiratory drive. Most diagnoses are made in pediatric patients, however late-onset cases have been rarely reported. Due to the milder symptoms at presentation that might easily go overlooked, these late-onset cases can result in serious health consequences later in life. Here, we present a case report of late-onset CCHS in an adolescent female patient. In this review we summarize the current knowledge about symptoms, as well as clinical management of CCHS, and describe in detail the molecular mechanism responsible for this disorder.

## 1. Introduction

Central congenital hypoventilation syndrome (CCHS), also known as Ondine’s curse, is a rare genetic disease. Most cases are sporadic, although it can be inherited in autonomic dominant manner with incomplete penetration. Up to date, only around 1300 cases worldwide have been reported [1]. It is most commonly caused by mutations in the Paired Like Homeobox 2B (PHOX2B) gene on chromosome 4 [1]. These mutations can be categorized into polyalanine repeat expansion mutations (PARMs) and non-PARMs (NPARMs), which include nonsense, missense and frameshift mutations [1]. PARMs are located in the second polyalanine repeat sequence in exon 3 of PHOX2B, and they are more prevalent than NPARMs [1]. Up to 50% of patients with CCHS also present with Hirschsprung’s disease (Haddad syndrome) [2]. CCHS is characterized by a lack of respiratory drive, manifesting primarily during non-rapid eye movement (NREM) sleep. Consequences of untreated CCHS include: hypercapnia, cor pulmonale and mild autonomous nervous system dysfunction [3]. Treatment is based on securing ventilation during sleep using non-invasive ventilation, such as bi-level positive airway pressure (BiPAP). Apneic pauses and related symptoms (e.g., cyanosis) are usually identified either by clinicians or parents during the neonatal period, which enables early diagnosis and the onset of treatment. In milder cases symptoms, are less pronounced and occur later in life. Thus, they frequently remain overlooked in adolescence. These are referred to as late-onset CCHS. The literature describes only 21 such patients; adolescent cases are even more scarce [4]. Here, we describe one such case.

## 2. Case Report

In 2003 a 14-year-old female was admitted to a Department of Cardiology with acute pericarditis. Medical history revealed that the patient was born at the 39th week of gestation with a low birth weight and congenital pneumonia (Apgar score: 5). She had no siblings. Treatment with continuous positive airway pressure was instituted during the first 24 h of life. Several hospitalizations due to upper or lower respiratory tract infections in the neonatal period, infancy and childhood had been recorded. Despite the respiratory failure with hypercarbia that occurred in their course, requiring periodic mechanical ventilation and apparent respiratory drive dysfunction, diagnosis of CCHS had not been established (according to the girl’s father; no medical charts were available).

At admission, electrocardiography (ECG) showed moderate hypertrophy of both ventricles. Echocardiogram confirmed the right ventricular (RV) hypertrophy and revealed fluid accumulation in the pericardial sack. Pulmonary hypertension, associated with RV hypertrophy, was present. Laboratory blood tests did not show any deviation from the norm, except for increased white blood cell count and C-reactive protein, indicating inflammation. Arterial gasometry results before and after 10 min of volitional hyperventilation changed significantly (PO^2^ from 69 mmHg to 82 mmHg, hemoglobin saturation from 93.2% to 97.6% and PCO^2^ from 54 to 25 mmHg). After the pericarditis was successfully treated with antibiotics (ceftriaxone during hospitalization, cefaclor after discharge), diuretics, nonsteroidal anti-inflammatory drugs, albumin transfusion, antiviral therapy and vitamin supplements, the patient was referred to the Respiratory and Sleep Disorders Centre. Spirometry, body plethysmography, and pulmonary diffusing capacity tests were conducted. The outcomes were normal, ruling out restrictive, obstructive or any other pulmonary diseases potentially affecting pulmonary function, as well as myopathies. Genetic tests of the patient’s and her parents’ PHOX2B gene were performed. The patient was heterozygous for PARM (20/25 PARM). Such changes were not present in the parents; thus, mutation was either de novo or a result of germline mosaicism. The diagnosis of CCHS, based on the observed periods of nocturnal apnea and desaturations, normal pulmonary function tests and PHOX2B mutation prompted BiPAP (Synchrony, Respironics, Murrysville, USA) treatment with nasal mask. Average volume-assured pressure support was used. Applied inspiratory positive airway pressure (IPAP) was in the range of 13–17 (average 16) and expiratory positive airway pressure (EPAP) was 5 mbar. Diagnostic polysomnography (PSG) after the onset of BiPAP treatment was not performed due to ethical considerations—weaning from therapy would have been necessary.

In 2014, the patient reported to the Centre with significant declines in oxygen saturation during sleep (down to 40%). The PSG was ordered to determine whether a change from the nasal to naso-oral mask was necessary. Despite using high pressure support (difference between IPAP and EPAP), long periods of saturation as low as 50% were observed during deep sleep, regardless of the patient’s position. Once the naso-oral mask was applied, saturation remained above 95% with no desaturations. There was no need for the adjustment of IPAP and EPAP pressures.

In 2018, the patient was referred to the Centre for PSG by a cardiologist due to nocturnal bradycardia (up to 28 events per min lasting up to 3000 ms) and non-sustained ventricular arrhythmia on 24 h ECG monitoring. A pacemaker implantation was considered. Spirometry, body plethysmography, and pulmonary diffusing capacity test results were unremarkable. The patient underwent a control PSG with BiPAP noninvasive ventilation (synchrony with average volume-assured pressure support). The minimal and maximal IPAP values were 12 and 18 mbar, respectively, and the average EPAP was 5 mbar. The tidal volume was set at 400 mL, with the respiratory rate at 14/min. Hemoglobin saturation was stable throughout the night, at an average of 97%, with the nadir of 93%, which confirmed the effectiveness of the BiPAP treatment.

In 2019 during another 24 h monitoring period, only 21 sinus pauses above 2 s without the atrio-ventricular block were registered. Our patient’s body-mass index was within or below normal through the whole follow-up period.

## 3. Genetic Causes of CCHS

The majority of CCHS cases are caused by mutations in PHOX2B at chromosome 4p12, as described in the introductory section of this article. The PHOX2B gene was identified in 1996 by Yokoyama et al. [5]. It is a transcription factor; a paralogue of PHOX2A [6]. Homodomain of this protein (domain that binds to the DNA regulating transcription) is similar to that of the proteins encoded by other homeobox genes. PHOX2B, among other functions, acts as a stimulator of the transcription of dopamine beta-hydroxylase and tyrosine hydroxylase through binding to the promotor [7]. Both enzymes are important to the biosynthesis of noradrenaline.

PHOX2B promotes cell cycle exit, neuronal differentiation and shows anti-proliferative activity [8,9]. Expression of PHOX2B might be only transient. In humans it is present primarily in the pons and the medulla, as well as in the medullary cells of the adrenal glands. Already in neonates, PHOX2B expression is seen only in the brain, not in the spinal cord, whereas during embryonic development it enables the development of branchiomotor neurons (cranial motorneurons) in the rostral cervical spinal cord and the hindbrain [8,10]. The PHOX2B gene is often co-expressed with PHOX2A. PHOX2B, similarly to PHOX2A, is present in the cranial sensory ganglia (VIIth, IXth, Xth ganglia) and all of the autonomic nervous system ganglia, from the beginning of the formation at least to the middle of the gestation period [10]. PHOX2B and PHOX2A might regulate each other’s expression. During embryonic development, in the dorsoventral axis and at rhombomeric borders, PHOX2B expression occurs before the PHOX2A [10]. However, in the cranial sensory ganglia, PHOX2A moderated the transcription of PHOX2B, as was evidenced in a study on mice [10]. PHOX2B is involved in the development of the noradrenergic neurons, thus facilitating the development of the locus coeruleus, the main source of noradrenaline in the brain, part of the reticular activating system and the nucleus of the solitary tract, which contains A2 noradrenaline synthetizing neurons [6,11]. It is also responsible for appropriate formation of the autonomic and enteric ganglia, as well as the central chemoreceptors and the carotid body (both locus coeruleus and the solitary tract are sensitive to the carbon dioxide) [6,11]. As already mentioned, through co-operation with *Nkx2.2*, mammalian achaete scute homolog-1 (Mash-1) Phox2b enables formation of the branchiomotorneurons [8].

**Figure 1:**
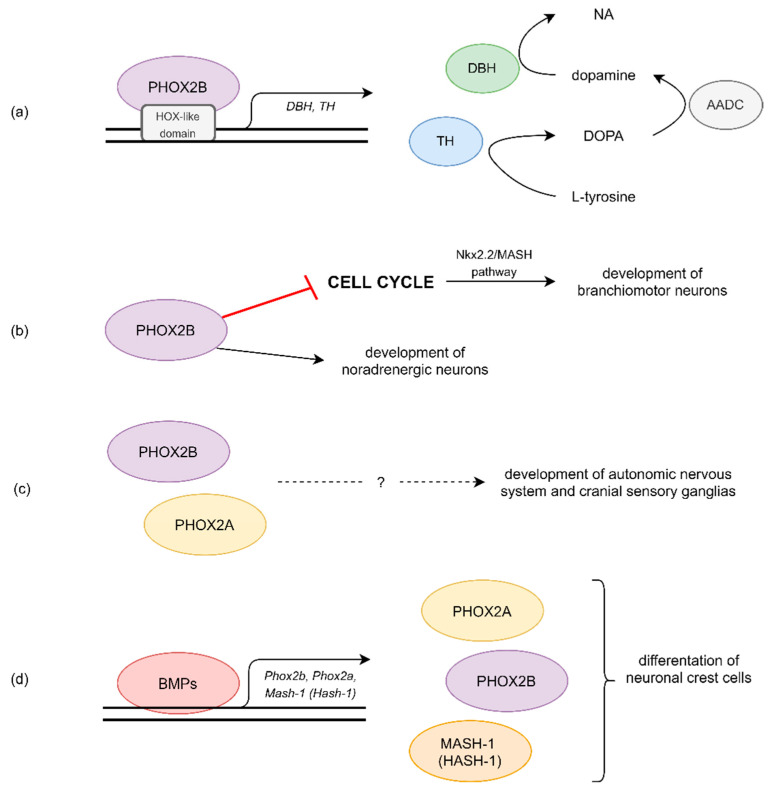
Possible pathomechanisms of CCHS. (**a**) HOX-like domain of PHOX2B binds to promotors of NA synthesis genes, such as DBH and TH, leading to their expression. (**b**) PHOX2B arrests some cell cycle in brain, what enables proper development of branchiomotor neurons. Similarly, it also has impact on development of noradrenergic neurons. (**c**) PHOX2A and PHOX2B expression was detected in the autonomic nervous system and cranial sensory ganglia of a fetus from the beginning of the formation at least to the middle of the gestation period. (**d**) BMPs promotes expression of neuronal crest cells differentiational factors, such as PHOX2A, PHOX2B and MASH-1 (HASH-1). AADC—dopa decarboxylase; BMPs—bone morphogenetic proteins; DBH—dopamine β-hydroxylase; HOX-like domain—homeobox like domain; MASH-1—mouse achaete-scute complex like 1 (HASH-1—human homologue of MASH-1); NA—noradrenaline; Nkx2.2—NK2 homeobox 2; PHOX2A—paired like homeobox 2A; PHOX2B—paired like homeobox 2B; TH—tyrosine hydroxylase.

Differentiation of the neural crest cells is stimulated by the bone morphogenic proteins, which activate transcription factors: PHOX2B, PHOX2A and MASH-1 (human orthologue of MASH-1 is HASH-1) (Figure 1) [6]. Thus, other genes might also be involved in the pathogenesis of CCHS. HASH-1 is transcription factor, responsible for the differentiation of olfactory and peripheral autonomic neurons. In vitro experimentation on mice showed that mutations of this gene might affect the development of the noradrenergic system [12]. In a study conducted by de Pontual et al., out of 30 patients with CCHS, three were heterozygous for a mutation in HASH-1 [12]. However, as the authors conclude, mutations in HASH-1 are not sufficient nor necessary to cause the CCHS phenotype [12].

Expression of both PHOX2B and PHOX2A is vital to the phenotype differentiation and development of the neurons and the hindbrain. PHOX2B facilitates the proper formation of structures associated with the autonomous nervous system, as well as other primitive functions, such as breathing. It can be regarded as the main regulator of noradrenergic phenotype differentiation. Aberrant formation of the noradrenergic system seems to be the main mechanism responsible for CCHS, however, there are other, not yet described factors affecting the disease onset and clinical presentation.

### 3.1. Breathing Regulation in CCHS

Control of breathing in humans is a complex process, involving the integration of information from the periphery and the central nervous system, with consideration for both automatic and volitional aspects.

In CCHS, the vast majority of patients experience problems with hypoventilation during sleep, and these are particularly pronounced in NREM. It is worth noting that REM does not promote apneic episodes, even though it is accompanied by the muscular hypotonia/atonia. PHOX2B gene expression appears to delineate a continuous neural circuit formed by the carotid body, afferent chemoreceptor neurons conveying excitatory input to the chemoreceptive neurons of the nucleus solitary tract projecting to the retrotrapezoid chemoreceptors and the ventrolateral medulla, which, in turn, regulate breathing [13]. Thus, researchers suggest that CCHS patients suffer from abnormalities in the chemoreceptive sites [14]. Both central and peripheral receptors are formed by cells originating in the neural crest. Central chemoreceptors are located on the ventral area of the medulla oblongata, close to the point of exit of the IXth and Xth cranial nerve. They include, for example, locus coeruleus, solitary nucleus tract, and retrotrapezoid nucleus; however, determining the exact structures responsible of chemoreception is difficult [15]. Carotid body, a peripheral chemoreceptor, might also be affected (as exemplified in autopsies of CCHS children) by the PHOX2B mutation, however, in animals with 27 poly-A expansions no abnormalities were found [16]. Thus, the main contributor to the pathogenesis of CCHS are central chemoreceptors.

Ventilation can be regulated by the VGlut2 (vesicular glutamate transporter 2) mRNA-containing neurons, which can be found in the ventral respiratory column in the retrotrapezoid nucleus. They show sensitivity to CO^2^ and promote breathing through glutamate release [17]. According to a study conducted by Dubreuil et al., mice with poly-A expansion in the PHOX2B gene (mutation occurring in the CCHS individuals), who died after birth due to a lack of respiratory drive had no VGlut2 in the rostral end of the ventral respiratory column [18].

Changes in breathing pattern accompany a stress response, regulated by the locus coeruleus. As was shown in a study in mice, PHOX2B-positive neurons in the locus coeruleus might increase ventilation, when stimulated, as well as increase the activity of the pre-Botzinger complex (main inspiratory oscillator), to which their axons project [19]. Since PHOX2B is required for the development of the locus coeruleus, mutant alleles might cause its dysfunction.

Characteristic of respiratory function changes are important to proper diagnosis and differentiation CCHS and lung diseases in early stage of diagnostics. Breathing disruption is a typical symptom for both disorders. In the case of lung diseases, respiration would be impaired both during wakefulness and sleep, as well as volitional and unconscious respiration, whereas CCHS has a clear pattern of the onset of symptoms during the night. Additionally, examinations, such us spirometry, body plethysmography, and pulmonary diffusing capacity tests, should be conducted, ruling out restrictive, obstructive or any other pulmonary diseases potentially affecting pulmonary function, what was occurred in the patient from the case report.

Respiratory support is a necessity in CCHS patients in order to avoid neurocognitive damage, especially during formative years. Possible therapeutic options include: positive pressure ventilation via tracheostomy, noninvasive positive pressure ventilation and diaphragm pacing [20]. The first method is usually applied in infants, and some authors believe that it allows for better oxygenation in the first few years in life compared with its noninvasive counterpart [20]. Noninvasive positive pressure ventilation can be initiated in older children and can continue throughout life, with devices, such as a nasal mask, nasal cannula or face mask [20]. It is recommended to use BiPAP as a continuous positive airway pressure; however, the standard therapy in OSA does not facilitate proper ventilation in this group of patients [20]. Diaphragm pacing, on the other hand, allows for weaning from mechanical ventilation altogether [21]. This aspect might be especially important, as it enables a child to freely explore the environment and interact with others, thus increasing life quality [21]. It is an invasive procedure; however, it has been proven to be safe and can be used in pediatric patients [21].

In summary, CCHS is a condition affecting a network of brain structures mediating chemoreception, as well as sympathetic and parasympathetic control. The majority of people afflicted with this disease require only nocturnal ventilatory support, however more severe cases might require constant ventilation. The particular treatment chosen for of therapy (i.e., invasive/noninvasive positive pressure ventilation, diaphragm pacing) should be appropriate for the patient’s age and needs.

### 3.2. Disorders Associated with CCHS

A combination of CCHS and Hirschsprung’s disease is called the Haddad syndrome. It was first reported by Haddad in 1978 [22]. It is a rare disorder, and up to 50% of patients with CCHS suffer from concomitant HSCR [2]. Haddad syndrome emerges as a result of a lack of parasympathetic mucosal or myenteric plexi in the distal hindgut. In consequence, muscles of the intestine are unable to relax, causing obstruction, which might be fatal, if left without treatment. It is a neurocristopathy, since cells colonizing the distal bowel are derived from the neural crest. In PHOX2B-deficient mice, progenitor cells from the vagal crest are able to descend into the foregut, however, the enteric nervous system is unable to develop. Haddad syndrome has been associated with NPARM and 20/27 PARM mutations [23].However, recently authors have been recommending screening for Hirschsprung’s all CCHS patients, regardless of the genotype [23]. The Haddad syndrome tends to be more severe in comparison with Hirschsprung, due to more extensive agangliosis, although total agangliosis occurs rarely. The most frequent appears to be agangliosis, going proximal to the sigmoid colon [6]. In addition, Hirschsprung’s disease occurs more often in males, with the male–female ratio being 4:1.14, whereas Haddad syndrome is as frequent in males as in females. CCHS patients also report other gastrointestinal symptoms, such as severe constipation. Here, the commonly accepted explanation is some sort of malfunctioning of the ganglia [24]. Esophageal dysmotility might also be present, although it tends to lose intensity after the first year of life [24]. The genetics of Hirschsprung’s disease are complex and are not yet fully understood. It has a non-Mendelian inheritance pattern with partial penetration. It might be a result of mutations in the Endothelin B receptor (EDNRB), Endothelin 3 (EDN3) or most commonly rearranged during transfection (RET). RET protooncogene encodes transmembrane receptors and members of the tyrosine protein kinase family of proteins [25]. It is a receptor for the following ligands: glial cell line-derived neurotrophic factor, neurturin, artemin, and persephin [26]. Being responsible for, among others, neuronal survival and differentiation, RET is necessary for the proper development of the enteric nervous system, as deletion results in complete agangliosis [26]. RET mutations causing Hirschsprung’s disease are usually loss-of-function mutations, present in 50% and 7–35% of hereditary and sporadic cases, respectively [25]. PHOX2B appears to influence the development of Hirschsprung’s disease through interactions with the RET promoter, although not directly; according to studies, there is no physical binding between them [25]. PHOX2B can transactivate the RET promoter through recruitment of other regulators, for example hairy and enhancer of split-1 (Hes1) and Kruppel Like Factor 4 (KLF4) [27]. According to Fernandez et al., poly-A expansions or other mutations causing flawed protein folding or oligomerization might be related to impaired function of the ventilatory neural circuit, whereas mutations where *PhoX2B* protein has no oligomerization properties (i.e., missense mutations, number of copies on genome) tend to strongly affect the enteric nervous system [27]. As one study conducted by di Zanni et al. shows, PHOX2B variants with poly-A contractions (deletions in the poly-A fragment) reduce its transactivational activity, thus leading to reduced RET expression, which results in the CCHS phenotype [25]. Overall, it can be hypothesized that NPARMs might exert more extensive pathogenic properties as they do not accumulate in the cell’s cytoplasmic compartments, like PARMs, but find their way to the nucleus. There, they affect downstream genetic circuits causing numerous aberrations [25]. As previously mentioned, HASH-1 might also be associated with Hirschsprung’s disease. In MASH-1-deficient mice, the enteric nervous system developed poorly in the bowels; moreover, serotonergic and esophageal neurons were absent [26]. However, this subject requires further study.

Autonomic nervous system dysfunctions might be present in individuals with CCHS to a variable degree. Ophthalmologic manifestations include: strabismus, anisocoria, and abnormal pupillary light response. Malfunctioning of the locus coeruleus might be linked to the abnormal pupillary light reflex response in patients with CCHS, since it is able to indirectly influence the pupil size. The exact mechanisms behind this process are still being discussed [28]. What is interesting, according to Patwari et al., is that there is an inverse linear relation between the pupil diameter, velocity and length of the poly-A sequence [29].

Other autonomous nervous system dysfunctions include congenital hyperinsulinism and the associated tendency to hypoglycemia. Fortunately, it is not a common finding. Origins of hyperinsulinism are not yet completely explained. It is evidenced that the neural-crest-derived PHOX2B-positive cells appear in the pancreas soon after they do in the stomach. These affect the development of beta cells: as one study shows, embryos deficient in PHOX2B had a higher amount of insulin-secreting beta cells [30]. This increase was caused by an increased cell proliferation rate, not neurogenesis [30]. Thus, it can be hypothesized that the aforementioned abnormalities in the pancreas development contribute to hyperinsulinism later in life.

Individuals with CCHS tend to have a slightly malformed facial structure with a broad, flat, rectangular face [31]. Lateral edges of the vermillion are turned inferiorly [31]. Such features were not present in our patient. Those characteristics are a result of impaired development of the dorsal rhombencephalon, in which PHOX2B is expressed [31].

Cardiac arrhythmias are common in CCHS; even though there are differences in their prevalence depending on the PHOX2B mutation type, researchers recommend screening to be a standard procedure in all patients [32]. Their origin is still a subject for discussion; however, it can be suspected that PHOX2B affects migration and maturation of the cardiac neural crest cells from the neural tube [32]. Cardiovascular manifestations of CCHS are not restricted to arrhythmias. In their cohort, Laifman et al. have found that CCHS patients tend to have a higher prevalence of anomalies affecting the proximal aortic arch and proximal coronary arteries [32]. Those structures also originate from the cardiac neural crest cells [32]. Impaired migration and differentiation of the cardiac neural crest cells is hypothesized to be a result of aberrant interaction between the SRY-related HMG-box (SOX10) and Twist-related protein 1 (TWIST1) [32]. Both act as transcription factors repressing PHOX2B expression through binding with the gene’s promoter [32]. PHOX2B mutations might disrupt this downregulation, but more research on the subject is needed in order to corroborate this hypothesis [32].

Mutations in the PHOX2B gene might also entail a higher risk for the development of tumors originating in the neural crest (neuroblastoma, ganglioneuroma, ganglioneuroblastoma). Their occurrence is much more common in individuals with NPARMs than those with PARMs, at 41–50% and 1%, respectively [4]. In neuroblastoma patients, PHOX2B mutations are infrequent. Thus, it can be hypothesized that missense or nonsense mutations in PHOX2B might act as an initiator to further events requiring other aberrations of the neural crest cells [33].

CCHS individuals might also experience seizures, usually resulting from hypoxia, hypoglycemia, arrhythmia and syncopes. Slight intellectual impairment might also be present, creating the need for education in special schools. Difficulties socializing or performing daily tasks also have been reported. CCHS patients tend to have reduced anxiety [34].

Body temperature dysregulation might be present in CCHS. According to Sayied et al., the mean peripheral skin temperature of those patients is lower than healthy controls and shows greater variability at night [35]. Researchers have proposed that such results show dysfunction in terms of control of the peripheral vascular tone [35].

CCHS is a neurocristopathy, manifesting primarily in the form of apneic episodes during sleep; however, as it is caused by mutation of gene important to the maturation and migration of neural crest cells, it might be accompanied by other symptoms. These include cardiovascular manifestations (bradycardia, prolonged sinus pauses, reduced heart rate variability, postural hypotension, etc.), profuse sweating and flawed body temperature regulation, slight cognitive impairment, inadequate perception of anxiety, and Hirschsprung’s disease.

## 4. Clinical Management of CCHS Patients

Perhaps the most important factor to consider for CCHS patients is ventilatory support. Weaning from the device is not to be anticipated, as CCHS is not curable [34]. In children using masks, it is important to be cautious and watch for signs of mid-face hypoplasia [36]. Special considerations should be given to the post-operative care of CCHS patients, as anesthetics might trigger apnea [34]. All patients should undergo a 72 h Holter ECG once a year to detect heart rhythm abnormalities, as they might require a pacemaker implantation [34]. Researchers recommend taking into consideration the diagnosis of Hirschsprung’s disease if a patient presents with suggestive symptoms, regardless of their age or the severity of their complaints [34]. Ocular examination should be performed on every CCHS patient at time of the diagnosis as well as the onset of symptoms associated with impaired sight [34]. Regular control of psychomotor and cognitive development is particularly important in children, where examination should be performed annually for the first few years of life [34]. CCHS patients, given that they are at a higher risk of developing neural crest derived tumors, should be controlled oncologically [34].

Novel approaches to treatment have emerged in the last decade. Interestingly, desogestrel (progestin drug) was shown to improve chemosensitivity in two female patients [1]. This finding was not replicated, although in our opinion it lays the groundwork for future research, as improving chemosensitivity would be beneficial for patients, especially in the long term [1]. Studies also focus on precluding damages done by the mutant PHOX2B through, e.g., folding, recovering or removal of protein through autophagy or modulating neuronal excitability through pharmacological targeting of potassium channels [1].

### Available Case Reports

As already mentioned, cases of CCHS in older children are extremely rare. One such family was described by Doherty et al. [37]. There, an 11-year-old male and his 14-year-old sister were diagnosed with CCHS [37]. Similarly to our patient, they had a history of respiratory tract infections requiring mechanical ventilation [37]. The male patient had a pulmonary hypertension (systolic pulmonary artery pressure 38 mmHg), an enlarged right heart, hypercapnia and hypoxia, although no heart rhythm abnormalities were detected on the ECG [37]. In this family, both patients and two younger children have inherited the PHOX2B PARM 20/25 mutation form their father in an autosomal dominant manner [37].

Al Rashdi et al. reported a case of a six-year-old female with 20/25 PARM [38]. In her case, late-onset CCHS seems to have been triggered by general anesthesia during dental extraction [38]. Attempts of extubation after the surgery were unsuccessful due to apnea [38]. The neonatal, infancy and childhood period was uneventful [38].

Diagnoses of CCHS in pediatric patients have been described by Herrera Flores et al., although here genetic analyses were not conducted and thus, they do not fulfill the criteria established by the American Thoracic Society [3,39]. In the first case, a 12-year-old male whose weight and height were under the third percentile was admitted to the hospital due to pneumonia [39]. Hypoxia, hypercapnia, RV hypertrophy and pulmonary hypertension (34 mmHg) were detected [39]. Apneic episodes during sleep were not confirmed even though he had severe hypercarbia while asleep [39]. Another patient was a 15-month-old male [39]. His development was age-appropriate, and the pregnancy was uneventful as well [39]. He was hospitalized due to pneumonia [39]. He developed severe hypercapnia and seizures [39]. Profuse sweating and heart rhythm abnormalities (brady/tachycardia) were identified [39].

Adult diagnosis of CCHS often seems to be incidental; for example, when CCHS diagnosis is confirmed in children (as was in the first mentioned case). Others might be triggered by surgery or there might be no known trigger at all [4]. Their symptoms might include: hypercapnia, hypoxia, RV hypertrophy, cyanosis, constipations, neurocognitive dysfunctions, headaches, and oedema [4]. Researchers also hypothesize that hypoxia might lead to miscarriage [4]. We summarized reported cases of late onset CCHS in the Table 1 Trochet et al. have described 17 more similar cases in their work from 2008 [40].

## 5. Clinical Implications

Our case shows that clinical vigilance is indispensable in order to diagnose early and treat late-onset CCHS, which was not the case in our patient. Disregard of the symptoms might lead to serious health consequences, resulting from hypoxia, hypercarbia and sleep apnea, such as pulmonary hypertension and RV hypertrophy. It is worth noting that hypoxia-related symptoms might also occur in certain myopathies (due to respiratory muscle weakness), and thus differential diagnosis is often needed [49]. Pre- and post-hyperventilation arterial blood gas tests, which were performed in case of our patient, might be utilized. As both CCHS and myopathic patients are able to voluntarily control their breathing, a significant improvement in oxygen blood level and decrease in carbon dioxide is expected, however in the latter group maximal ventilation should cause respiratory muscle fatigue. Here, the patient’s parents also have reported long periods of sleep apnea and cyanosis without visible respiratory muscle excursions.

CCHS patients are at a higher risk of autonomic heart rhythm dysfunction or arrhythmias, which might be associated with PHOX2B gene mutations and following hypothalamic functional abnormalities [50]. However, they can also be complications of hypoxemia and hypoventilation, such as in obstructive sleep apnea, where arrhythmic episodes increase with hypoxia severity and the number of apneic events [51]. Heart rhythm dysfunctions might be life threatening, thus clinicians should be able to early detect and address those issues.

Regular follow-ups with saturation assessment and mask adjustments are particularly important in adolescents with CCHS, as facial morphology might change with puberty. In our patient, severe hypoxia has developed even though the adolescent had reportedly good compliance. Several possible causes of hypoxia, i.e., mouth breathing, obstruction of the upper airways and mid face hypoplasia following mask use, were ruled out on examination. Oral leak was present, and switching from a nasal to naso-oral mask resolved the issue.

Post and perioperative complications should be taken into account in the anesthesia of CCHS patients. In our case, an otherwise unexplained apneic episode occurred following pethidine administration. It does not appear to be an isolated case; CCHS patients might be susceptible to respiratory depression after opioid administration [52]. In their systematic review, Basu et al. recommend considering CCHS in the differential diagnosis after such an event [52].

## 6. Conclusions

Late onset CCHS is a rare, possibly underdiagnosed condition that requires a complex management. In patients with mild symptoms, it might remain undiagnosed until consequences of sleep disturbances, such as cor pulmonale, emerge. However, adequate treatment, introduced at an early stage allows individuals with late onset CCHS to lead a normal life, avoiding destructive complications of chronic hypoxia.

## Figures and Tables

**Table 1:** Late onset pediatric central congenital hypoventilation syndrome, literature review.

No.	Author	Sex	Age	Triggering Factor	Clinical Presentation	PHOX2B Mutation	Heredity	Treatment
**1.**	Cohen-Cymberknoh et al. [41]	F	12 y.o.	Respiratory infection	Hypercapnic respiratory failure requiring mechanical ventilation. Occasional headaches.	20/25 PARM	NA (Father unavailable)	NIV during sleep
**2.**	Mahfouz et al. [42]	F	6 y.o.	Anesthesia	Intractable postoperative hypoventilation.	20/25 PARM	NA	Mechanical ventilation via tracheostomy during sleep
**3.**	Jennings et al. [43]	M	26 m.o.	Respiratory infection	Hypoxemic episodes during sleep, recurring respiratory infections requiring hospitalization. Mediastinal ganglioneuroblastoma.	0.7 Mb deletion of PHOX2B and other genes	Maternal	NA
**4.**	Parodi et al. [44]	M	15 m.o.	Respiratory infection	Hirschsprung’s disease, hypotonia. Respiratory infections requiring mechanical ventilation. Hypercapnic respiratory failure.	missense mutation in exon 2 c.419C > A	De novo	NIV during sleep
**5.**	Doherty et al. [37]	M	11 y.o.	Respiratory infection	Respiratory infections requiring mechanical ventilation. Repeating hypercapnic respiratory failure. Pulmonary hypertension.	20/25 PARM	Paternal	NIV during sleep
**6.**	Doherty et al. [37]	F	14 y.o.	Respiratory infection	Respiratory infections requiring mechanical ventilation. Repeating hypercapnic respiratory failure.	20/25 PARM	Paternal	NIV during sleep
**7.**	Al Rashdi et al. [38]	F	6 y.o.	Anesthesia	Intractable postoperative hypoventilation.	20/25 PARM	NA	Mechanical ventilation during sleep
**8.**	Repetto et al. [45]	M	2.75 y.o.	Anesthesia	Intractable postoperative hypoventilation.	20/24 PARM	Maternal	Mechanical ventilation via tracheostomy during sleep
**9.**	Magalhaes et al. [46]	F	13 m.o.	Respiratory infection	Hypercapnic respiratory failure. Respiratory infections requiring mechanical ventilation.	Nonsense mutation in exon 1 c.23dupA	De novo	NIV during sleep
**10.**	Chuen-im et al. [47]	F	3 y.o.	Respiratory infection	Hypercapnic respiratory failure. Cor pulmonale.	20/24 PARM	Maternal	Mechanical ventilation during sleep
**11.**	Fine-Goulden et al. [48]	F	12 y.o.	Anesthesia	Hypercapnic respiratory failure. Cor pulmonale. Mild learning difficulties. Dyspnea on exertion. Fear of falling asleep, somnolence, sleep fragmentation. Cyanosis.	20/25 PARM	NA	Mechanical ventilation during sleep
**12.**	Herrera-Flores et al. [39]	M	12 y.o.	Respiratory infection?	Hypotrophia. Cor pulmonale. Severe chronic respiratory insufficiency.	Genetic analysis was not performed	NA	The patient died of cor pulmonale and respiratory failure 6 months later
**13.**	Herrera-Flores et al. [39]	M	15 m.o.	Respiratory infection	Hypercapnic respiratory failure. Episodes of tachy/bradycardia. Diaphoresis. Mild enlargement of the right heart.	Genetic analysis was not performed	NA	NIV with respiratory rate support

Abbreviations: F—Female, M—Male, m.o.—months old, NIV—non-invasive ventilation, PARM—polyalanine repeat expansion mutations, y.o.—years old.

## Data Availability

Not applicable.
